# A Holistic Model of Care to Support Those Living with and beyond Cancer

**DOI:** 10.3390/healthcare4040088

**Published:** 2016-11-18

**Authors:** Tamara Cadet, Cindy Davis, Jacinta Elks, Patricia Wilson

**Affiliations:** 1 School of Social Work, Simmons College, 300 The Fenway, Boston, MA 02115, USA; Tamara.cadet@simmons.edu; 2 Oral Health Policy and Epidemiology, Harvard School of Dental Medicine, Boston, MA 02115, USA; 3 Faculty of Arts, Business & Law, University of the Sunshine Coast, Sunshine Coast, QLD 4556, Australia; 4 School of Nursing, University of the Sunshine Coast, Sunshine Coast, QLD 4556, Australia; jelks@usc.edu.au; 5 Bloomhill Cancer Care, Sunshine Coast, QLD 4556, Australia; patricia@bloomhill.com.au

**Keywords:** cancer, survivorship, holistic care, model, Australia

## Abstract

Background: Globally, the burden of cancer continues to increase and it is well-documented that while not a homogeneous population, cancer patients and cancer survivors face many physical, psychological, social, spiritual, and financial issues. Cancer care is shifting from a disease-focused to a patient-centered approach resulting in an increased need to address these concerns. Methods: Utilizing a quality improvement approach, this paper describes an integrated cancer care model at Bloomhill Cancer Center (BCC) in Queensland, Australia that demonstrates the ability to meet the holistic needs of patients living with and beyond cancer and to identify opportunities for better practice and service provision. Results: Survey results indicate that 67% and 77% of respondents were very satisfied and 27% and 17% were satisfied with their first contact and very satisfied with their first meeting with a nurse at BCC. Clients also reported being very satisfied (46%) or satisfied (30%) with the emotional support they received at BCC and over 90% were very satisfied or satisfied with the touch therapies that the received. Conclusion: Due to the early success of the interventions provided by BCC, the model potentially offers other states and countries a framework for supportive cancer care provision for people living with and beyond cancer.

## 1. Introduction

Globally, the burden of cancer continues to increase with an expected 19.3 million new cancer cases by 2025. In 2012 (the most recent data available), 14.1 million new cancer cases were diagnosed and 8.2 million people died from cancer [1,2]. The US expects that in 2016, there will be 1,685,210 new cancer cases and 595,690 cancer deaths [3]. In Australia/New Zealand, there were an estimated 143,500 individuals diagnosed with cancer and 52,000 who died from cancer [4]. Worldwide, there are 32.6 million five-year cancer survivors including Australia [1,2]. In Australia, more than 66 per cent of cancer patients in Australia will survive at least five years after diagnosis. The most recent data indicates that there are approximately 800,000 cancer survivors in Australia [5]. Cancer survivors in Australia represent approximately 3%–4% of the total population. It is well-documented that while not a homogeneous population, cancer patients and cancer survivors face many physical, psychological, social, spiritual and financial issues [6,7,8]. In fact, cancer care is shifting from a disease-focused to a holistic approach, in which more attention is paid to psychosocial aspects, quality of life, patients’ rights and empowerment and survivorship [9,10]. As a result, it is critical to increase efforts to address these concerns [8,11].

### 1.1. Holistic Cancer Care

Providing holistic care is a component of providing integrative care [12,13]. Integrative care utilizes conventional and complementary approaches to cancer integrating them with a focus on the whole person, which includes emotional, spiritual, social, and lifestyle (diet, physical activity, sleep, relationships) factors, the provider-patient relationship as a partnership and interprofessional collaboration [14,15,16]. Some of the principles of an integrated care model include:

- Integrated care models emphasize a patient centered approach;
- Services are provided in a consistent approach across a range of providers, not just one organization or provider;
- Patients should have access to high quality care as close to home as possible assuming the availability of equipment and resources exist;
- Shared protocols, guidelines, and care pathways ensure consistency of care;
- Comprehensive screening for supportive care needs is critical to ensuring holistic care for cancer patients [17]

Responding to the individual needs of patients in a holistic and patient-centered manner is extremely important living with and beyond cancer [18,19,20]. Holistic care that looks past the physical needs, such as recovering from the effects of cancer treatment and addressing the risk factors associated with recurrence [8,21] and incorporates the other needs of the body and spirit, is a critical unmet need [22]. Cancer patients and survivors are often uncertain about next steps in their cancer journey and holistic care becomes an increasingly important approach [6]. Despite the evidence to support holistic care, empirical research indicates a lack of evidence regarding post-treatment services [23,24]. Patients frequently report that their psychological and other supportive care needs are neither identified nor addressed [24,25,26,27]. Furthermore, cancer survivors do not consistently receive survivorship care plans (SCPs) [24]. The SCP is an individualized document providing a summary of the patient’s diagnosis and treatment plan, possible side effects, healthy lifestyle habits, and available resources for financial and psychosocial support [28]. Increased research is greatly needed to expand the evidence base required to define optimal care delivery, including the type or components of care delivered, the manner in which that care is delivered and by whom, and the efficacy of the various models of care [8].

### 1.2. Survivorship Care in Australia

Although there is very active survivorship research in Australia, work to improve survivorship care to date has been patchy and not coordinated [29]. Various models of care exist across the states of Australia from the disease specific model, general survivorship model, consultative clinic, integrated care model and transition to primary care model resulting in state-specific frameworks, practice guidelines and services [30]. There are differences in service offerings and utilization between those who live in highly populated areas and those who live in non-metropolitan cities and towns, including rural and remote locations [31] resulting in disparities. Close to 33% of Australia’s population live in regional and remote areas [32]. Furthermore, research indicates that survivorship care plans (SCPs), which offer an opportunity to meet survivor’s needs outside of a focus on cancer recurrence, are not used consistently in Australia [29]. Increasing the capacity to better care for patients in regional cancer centers has been proposed as one possible solution to address disparities [31,33]. The aim of this paper is to describe a model of supportive care for cancer patients and survivors in regional Queensland Australia. This may provide insight for other care providers to consider supportive cancer care models for patients and survivors that encompass principles of holistic care. Because much of Australia’s health care services are delivered at the State level, research is most feasible at the state level. Queensland, Australia, as a state provides an exemplar for others to consider because of some similarities in healthcare models and in populations [34]. Recent data suggests that the overall cancer survivor rate for the Queensland population has increased over 30% over the last twenty years [35] with more than 200,000 survivors at the end of 2012 who had been diagnosed with an invasive cancer since 1983 [36,37].

## 2. Methods

Using a quality improvement approach, this paper seeks to describe how the Bloomhill Cancer Center (BCC) utilizes the evidenced based principles of holistic care described above to explore patients self-reported experiences and expectations of care living with and beyond cancer and to identify opportunities for better practice and service provision. The philosophy and services of BCC are presented followed by the results of a quality improvement client survey of needs.

### 2.1. Design

This investigation utilized an observational non-equivalent group comparison design from a retrospective survey of all eligible patients. The survey was developed by one of the authors in consultation with the staff at BCC. The survey included quantitative questions on a 5 point Likert scale ranging from “very dissatisfied” to “very satisfied” on topics such as their first visit, contact with oncology nurse, touch therapies and emotional support. In addition, qualitative questions were used to elicit further information to validate the quantitative responses. Questions were also included to evaluate patients’ future needs for services, such as, retreats, yoga, nutrition information, managing the impact of hormone therapy, and financial/legal services.

A Client Survey of Need was mailed to a randomly generated cohort of 100 clients active on the Bloomhill HMS database in the first week of April 2015. Stamped self-addressed envelopes were enclosed to facilitate survey return. Thirty-five surveys were returned in total, giving a higher than average response rate. Data suggest that average return rates for surveys of this nature are between 25% and 30% [38]. Given the nature of the survey, descriptive statistics were used to analyze the data.

### 2.2. Model of Care

Bloomhill Cancer Care (BCC) is an integrated model providing holistic care [35,39]. BCC is a recognized center of excellence for integrated cancer care, balanced by their connection with and understanding of their clients and their needs [40]. BCC supports people of all ages on the Sunshine Coast, a rural/regional one hour from Brisbane, Australia, to live well with cancer. BCC is an independent, not-for-profit organizational supported by a professional team of approximately 30 staff and contractors and over 400 volunteers. The team consists of nursing and allied health practitioners who work closely with clients’ general practitioners, cancer specialists, nursing services, and other health care providers to create a network of care for their patients. BCC operates in partnership with the University of the Sunshine Coast to support quality service provision and receives no ongoing government funding to provide services. In fact, their services are funded through the proceeds from their Op Shops located across the Sunshine Coast, Queensland, Australia. BCC makes every effort to minimize the financial impact on people affected by cancer. Nursing, counseling, and volunteer services and social groups are all provided free of charge. BCC offer an initial Welcome Pass which provides free access to group service for a period of three months, plus an initial complimentary touch therapy session. Exercise, yoga, meditation, and lymphoedema management classes are all covered by a Session Pass for $50 for 3 months access or $10 fee for each service. Touch therapies and acupuncture have co-payments of $40 and $50 respectively and transportation costs vary relative to the mileage traveled. However, no one is denied services due to inability to pay.

### 2.3. BCC Philosophy

BCC treats the whole person by supporting the principles of empowerment and independence in all interactions with clients—which contributes to their connection and understanding of their clients and their client’s needs. BCC aims to enhance the quality of life for people living with and beyond cancer, through practical, physical, and emotional support utilizing education and awareness. With their core values of respect, innovation, professionalism, integrity, confidentiality, and leadership, BCC not only understands that health is more than the absence of illness but actively offers support across the six dimensions of wellness, that include physical, emotional, intellectual, social, spiritual, and occupational [41].

### 2.4. Description of the Model

**Figure 1:**
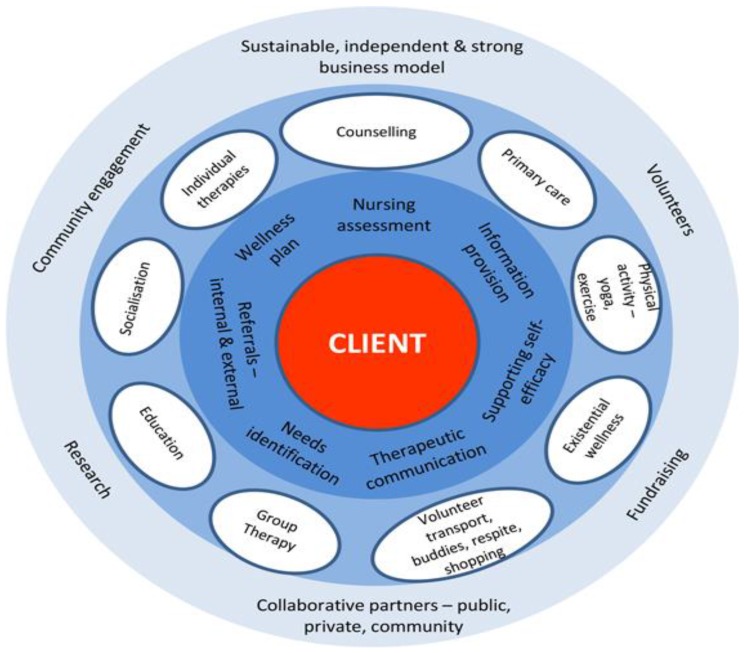
Bloomhill Cancer Center (BCC) Integrated Cancer Care Model.

BCC Integrated Cancer Care model as depicted in Figure 1 positions the client in the center of the services. The nursing team consists of skilled cancer care nurses who offer coordination of care through assessment, support, identification of needs and referral to appropriate services. The focus of the nursing service is to enable and empower clients to successfully manage their health and wellbeing. Nursing services assess clients holistically and obtain a health history to develop a personalized Wellness Plan using BCC, community and other health care resources to best support clients. The cancer nurses assist in the management of a range of cancer related issues including physical symptom management, understanding of the cancer trajectory, treatment options, and treatment decision-making support. Other services provided include survivorship care planning, support with cancer related sexual dysfunction and adjustment to hormone therapy or treatment induced menopause. Psychology services offer evidence-based psychological care and interventions provided by certified counselors and/or clinical psychologists. Dietician services provide dietetic and nutritional advice through both individual consultations and group activities. Due to the increased understanding of the role of exercise in positively impacting anxiety, fatigue, nausea, and prevention of cancer recurrence, exercise physiology classes are provided in addition to individual consultations to develop personalized exercise programs. Support groups, facilitated by cancer nurses and counseling staff, are based on supported social interaction providing an opportunity to meet with others who may be sharing a similar journey. BCC provides opportunities for personalized support for more distressed clients. Some of BCC Services include:

- Ongoing support from experienced oncology registered nurses;
- Access to support and information from cancer clinical nurse specialists;
- Support groups for the patient and carer (e.g., Living with Cancer treatment, Chemo Brain support group);
- Qualified Allied Health Care, including clinical psychology, counsellors, dietician, and exercise physiology services;
- Free counselling services;
- Lymphoedema management; and Complementary services (e.g., Touch therapies, Meditation, Mindfulness, Yoga).

Volunteers are responsible for facilitating art groups, transporting patients to medical appointments, providing respite care, providing buddy support for companionship to the patient or caregiver which can include sharing a cup of coffee, or going for a walk, shopping and meal preparation services.

### 2.5. Child and Adolescent and Young Adult Services

Recognizing the developmental, physical, emotional and social differences that exist between adults and children, adolescents, and young adults, BCC, consistent with its philosophy of creating personalized plans for patients and survivors, has specific approaches to practice with children, adolescents, and young adults. Utilizing a holistic approach to the care of the child client, a team approach builds resilience across the family support structure of the child. The Pediatric Psychology Preventative Model [42] is the model to guide the psychosocial assessment. The primary goal of a psychosocial assessment attempts to gather information regarding a young cancer patient and/or their family’s adaptation to the disease. This adaptation is an ongoing process of adjustment [43]. BCC utilizes the Clinical Oncology Society of Australia’s Guidelines for Psychosocial Management of Adolescents and Young Adults (AYAs) diagnosed with Cancer: Guidelines for Health Professionals [44] in its practice with AYAs. BCC is continuing to develop other AYA initiatives with its partners to provide activity based quarterly support group (PlayStation, pizza, volleyball), and tailored individual counseling opportunities.

## 3. Results

### Results of Client Survey of Needs

Patients were the primary responders at 65% followed by caregivers at 23%, family members and bereaved members at 3% and 9% respectively. The majority of respondents (56%) were older than 60 years of age followed by 41% between the ages of 46–60 years of age. Eighty-three percent were female, and about half (49%) had completed treatment. The most common referral path for clients to BCC was from a friend (49%) followed by referral from medical professionals (23%). Literature indicates that patients’ experiences in healthcare settings often start with their first contact [45]. Findings from this survey indicate that more than two-thirds (67%) of respondents were very satisfied with their first contact. Most people telephoned and made an appointment, but a third arrived without notice. Qualitative comments validated these finding with comments including: “Front desk very sympathetic and organized help quickly” and “Reception staff delightfully warm and friendly”. More than three-quarters (77%) of respondents were very satisfied with their first meeting with a cancer nurse specialist. Respondents reflected the level of satisfaction reported by respondents including: “My nurse listened and let me ‘cry it out’. Encouraging and discussed availability of assistance at Bloomhill” and “Amazing—she had experienced cancer too and was very understanding and informative about many issues”. Clients were very satisfied (46%) or satisfied (30%) with the emotional support they received at BCC and over 90% were very satisfied or satisfied with the touch therapies that the received. Respondents validated these findings with comments such as: “Loved my massages!” and “My massage therapist was like an angel and incredibly helpful”.

Respondents were asked about their need for further information and the top three needs were more information on mindfulness and meditation, managing fear of recurrence and releasing stress/renewing energy. About one-fourth of respondents wanted nutrition information, assistance with the emotional effects of cancer, and help getting back to normal. Overall, while BCC ranks high in client satisfaction with clients, they recognize areas of improvement and strive for their care process to be client-centered rather than service-centered. BCC want effective and warm communication in every client interaction which is demonstrated by providing explanations of what is happening and what to expect to support clients to respond positively to services [45]. Some qualitative comments are: “Wonderful services provided”, “Information to do with Bloomhill should be available in medical places”, “Without Bloomhill my health would not be as good as it is today”, and “Bloomhill is how I imagine Heaven to be!”

## 4. Discussion and Conclusions

This aim of this investigation was to describe how the BCC utilizes principles of holistic care to explore patients self-reported experiences and expectations of care living with and beyond cancer and to identify opportunities for better practice and service provision. While this paper provides formative data to BCC for improvement, it also provides promising evidence in support of models that provide holistic care for those living with and beyond cancer. There are several limitations in this study that should be noted. First, there was a small sample suggesting caution when interpreting the results. Furthermore, while patients were not required to participate, those who choose to participate self-selected, which could bias the sample. Specifically, there may be a difference between those who choose to participate and were satisfied with the services versus those who were not satisfied with services and choose not to participate. Finally, there was not a comparison group so we do not have data to determine the differences between those who responded to the survey and those who did not. Despite these limitations, this study provides a contribution to the emerging literature that discusses the utilization of holistic care for patients living with and beyond cancer. Furthermore, this study offers potential support for further research to influence future policy and funding decisions.

While BCC strives to be a center of excellence for integrated cancer care, there are challenges to address to ensure that BCC not only maintains its profound connection and understanding of their clients and their needs, but is able to directly affect those clients who continue to be underserved in their region. The primary challenge is to meet the needs of the growing number of cancer survivors within rural and regional areas. Many survivors and their families are not seeking available and needed services. Patients and family members are often overwhelmed at the time of diagnosis and treatment when referrals are made to supportive care services, such as BCC. Thus, they do not take advantages of available services to assist them during and after treatment. As a result, BCC plans to expand services in rural areas to increase the likelihood of access. With services available in those areas, BCC can work to help families seek services. Furthermore, there is a dominance of breast cancer services offered in Queensland and Australia [46]. Therefore, reaching other populations such as men is difficult. As a result, BCC is planning to offer services specifically for men including a support group and exercise classes and BCC networks with other cancer organizations such as prostate cancer groups and the Cancer Council. Finally, while funding is an ongoing challenge for BCC, BCC is able to rely on grants, community support, fundraising and their Op shops and not on government funding. The strength in not using government funding provides BCC opportunities to be creative and respond quickly to meet the needs of their clients. Further research to gain greater clarification on the facilitators and barriers to sustaining the program is important. BCC has a continuous quality improvement strategy to ensure that they meet their mission and goals. Continuous research and evaluations of the program will help in this process. As a community based organization, the evaluation of services and the delivery of evidence-based intervention is a central focus to ensure quality services and sustainability. In order to develop a systematic approach to evaluation, the BCC has formed a strategic alliance with the university to assist with the implementation of a rigorous evaluation and ongoing monitoring of patient outcomes. This will provided much needed outcome based data on the provision of services within a holistic integrated cancer care setting and ensure that the serviced being provided to clients are evidence based interventions and help to secure non- governmental grants and support. Future research will examine specific measures of quality of life to understand improvement in the clients’ lives, gaining the perspectives of the team working with patients, with the goal of demonstrating the effectiveness and possible replication of the program to other remote and regional areas of Australia and beyond.
